# In-Flight Tests of Intruder Detection Vision System

**DOI:** 10.3390/s21217360

**Published:** 2021-11-05

**Authors:** Paweł Rzucidło, Grzegorz Jaromi, Tomasz Kapuściński, Damian Kordos, Tomasz Rogalski, Piotr Szczerba

**Affiliations:** 1 Department of Avionics and Control, Faculty of Mechanical Engineering and Aeronautics, Rzeszow University of Technology, al. Powst. Warszawy 8, 35-959 Rzeszów, Poland; d.kordos@prz.edu.pl (D.K.); orakl@prz.edu.pl (T.R.); psz@prz.edu.pl (P.S.); 2 Eurotech sp. z o. o., ul. Strefowa 3, 39-300 Mielec, Poland; g.jaromi@eurotech.com.pl; 3 Department of Computer and Control Engineering, Faculty of Electrical and Computer Engineering, Rzeszow University of Technology, W. Pola 2, 35-959 Rzeszów, Poland; tomekkap@prz.edu.pl

**Keywords:** intruder detection, vision system, airspace, aircraft, airplane, in-flight tests

## Abstract

In the near future, the integration of manned and unmanned aerial vehicles into the common airspace will proceed. The changes taking place mean that the safety of light aircraft, ultralight aircraft and unmanned air vehicles (UAV) will become an increasing problem. The IDAAS project (Intruder Detection And collision Avoidance System) meets the new challenges as it aims to produce technically advanced detection and collision avoidance systems for light and unmanned aerial vehicles. The work discusses selected elements of research and practical tests of the intruder detection vision system, which is part the of IDAAS project. At the outset, the current formal requirements related to the necessity of installing anticollision systems on aircraft are presented. The concept of the IDAAS system and the structure of algorithms related to image processing are also discussed. The main part of the work presents the methodology developed for the needs of dedicated flight tests, its implementation and the results obtained. The initial tests of the IDAAS system carried out on an ultralight aircraft generally indicate the possibility of the effective detection of intruders in the airspace with the use of vision methods, although they also indicated the existence of conditions in which this detection may prove difficult or even impossible.

## 1. Introduction

Air traffic has increased significantly on a global scale in recent times. Simultaneously many local airports have achieved a several-fold increase in the number of flight operations. Unfortunately, on the other hand, quite a significant number of less well-trained amateur pilots have appeared, contributing to an increased number of incidents in aviation. In the near future, the integration of manned and unmanned aerial vehicles in the common airspace is expected. The official document [1] presents the roadmap for the integration process of both manned and unmanned air traffic in the European airspace. Some practical possibilities in this field were presented in several scientific and research papers [2,3,4,5]. The authors of [2] propose using the standard ADS-B (Automatic Dependence Surveillance-Broadcast) systems for RPAS (Remotely Piloted Aircraft Systems) separation. The works [3,4] concern an overall approach to a problem of collision avoidance by unmanned systems. From the point of view of the subject matter presented in this article, the approach presented by the authors of [5], who are considering the use of vision systems for visual flight rules-based collision avoidance, is very interesting.

The changes which are currently taking place in the structure of air traffic, make the safety of light aircraft and ultralight aircraft an increasing problem. New works on novel anticollision systems are currently being carried out in many centers around the world. Standard ACAS (Airborne Collision Avoidance System) class anticollision systems [6], such as TCAS (Traffic Alert and Collision Avoidance System) [7], require the use of SSR (Secondary Surveillance Radar) transponders and directional antenna systems [8]. TCAS is an airborne traffic alarm and collision avoidance system which is independent of air traffic control on the ground [9]. TCAS is not always applicable for small (and even more so ultralight) aircraft, mainly due to their mass and dimensions, as well as high installation costs. ADS-B is more affordable and can be used for both auxiliary traffic collision avoidance and surveillance in the airspace system. The possibilities of data fusion from TCAS and ADS-B systems are also very promising [9,10]. Due to the costs of purchase, installation and maintenance, but also due to the specificity of the intruder detection and avoidance algorithms required in small aircraft [11], sailplanes, and RPAS/UAV (unmanned aerial vehicles) [12,13], other systems have also been developed. The FLARM (flight and alarm) system is widely distributed in the world of aviation and is dedicated for sailplanes and UAV oriented applications [14]. FLARM uses technologies related to radio communication and satellite positioning. In addition to technologies related to radiolocation [14,15,16,17], also optical rangefinders [18], vision systems [19,20], and even audio detection systems [21,22,23] can be used to detect intruders and thereby avoid midair collisions. Two main type of “sense and avoid” vision systems can be distinguished: infrared camera systems and daylight camera systems [24]. In recent years, several concepts of integration of telecommunication, radar, navigation, vision, and audio subsystems into one complex “sense and avoid” system have also been promoted [25,26,27,28].

The IDAAS (Intruder Detection And collision Avoidance System for light aircraft) project that this work relates to, assumes the integration of independent sensors as well as the implementation of a compact measuring and computing module that works directly with on-board systems [28,29]. It is assumed that the anticollision system is developed as configurable in terms of the number and quality of sensors in relation to the characteristic target air platforms. The main strength of the IDAAS system will be full autonomy and independence from other devices and systems. It can be used as an independent primary or auxiliary anticollision system, according to the formal requirements defined in documents [30,31]. Up to now, various types of subsystems and sensors have been investigated, with a particular focus on vision-based varieties. The authors’ previous experience in this field [32,33,34,35] is also taken into consideration in the presented research.

## 2. Algorithm for Intruder Detection Vision System

The intruder detection algorithm applied in the developed system uses an analytical method to determine the optical flow of image points. Our approach is due to the stability and wide range of applications of this method described in the literature. For example, article [36] considers using optical flow techniques to detect objects for fluvial hyperspectral imagery. The method proposed by the authors provides a significant reduction of spatial errors compared to template matching. The presented study considers calculating the optical flow velocities from RGB and hyperspectral images. The first type of data for optical flow is extracted from RGB images after applying the Harris Corner detection algorithm. The second is extracted directly from hyperspectral images. In the article [37], the authors rightly point out the importance of the optical flow techniques in UAV vision systems as well as presenting some imperfections of optical flow in certain cases such as the presence of obstacles in the form of walls. The authors proposed a technique that is useful in detecting these types of obstacles in 3D environments. The studies revealed differences in the optical flow results depending on the object’s orientation relative to the observer, and helped to determine the proper MAV (micro aerial vehicle) obstacle avoidance strategy. The presented hybrid avoidance strategy is intended to detect close obstacles in 3D space and so, the proposed approach can be applied in enclosed, near-ground areas restricted by obstacles like building walls, sound-absorbing walls, protruding elements of buildings, etc. In the article [38], the authors describe using optical flow to recognize moving objects, presenting results of land vehicles detection for complex backgrounds. Their studies show the comparison of Horn–Schunck, Lucas–Kande, and FDRIG optical flow methods. The last of these methods is based on the special warping algorithm. Currently, we are seeing some trends in the development of vision systems using artificial intelligence. Such an approach is presented in [20], wherein the authors analyze the similar application of a vision system like ours but based on deep learning neural networks. The presented method discusses an aircraft multistage detection system based on such an approach. In the first stage, the background image is pre-processed. Then, in the next stage, the aircraft is detected by temporal filtering. Overall neural-type approaches seem to be very effective and interesting, furthermore, they follow one of the trends of vision systems development. Artificial intelligence gives very satisfying results, increasing the speed of processing and robustness. On the other hand, it requires a sufficient amount of video material and proper training techniques. These techniques allow the recognition of the characteristic features in an image, e.g., airplane wings, fuselage fragments, etc. Generally, there may occur some limitations when a potential intruder object is so far away from the observer that these features cannot be clearly extracted from an image. The authors in [20] mention that there were some problems in the case of multiple aircraft detection using the proposed approach, however the training data used may be responsible for this. Unfortunately, in the developing process, neural techniques are time-consuming and require a large amount of representative database material for training. It is particularly important in such aviation systems because it requires a lot of in-flight tests, which are expensive and risky. In our approach, the optical flow was most convenient and reliable because it takes into account some initial assumptions. The basic assumptions of the adopted method of optical flow are as follows [36,39]:

- The constant brightness of moving points.
- Small movement of each analyzed point.

These assumptions seem to be valid for the task of detecting moving objects as well as phenomena appearing far from the camera. Thus, the developed anticollision system is capable of detecting intruders when they are still at a safe distance. Finally, threatening objects can be detected as early as possible, providing a pilot or a flight control system with enough time to take proper anticollision action. The method is especially intended to detect objects that are moving relative to the background, however, it is possible to detect objects that are static to the background but are approaching or moving away from the observer. These types of objects, although remaining stationary against the background, change their geometric and dimensions (they increase faster the closer they are). In such cases, the algorithm can detect them and still give a chance to avoid collision. Theoretically, it is possible that objects are static to the background and do not change their size. That would mean they are moving at a similar speed and flight direction to the observer, and they do not pose a serious threat.

The main part of the algorithm is based on the optical flow, which is possible to apply thanks to the brightness adaptation module. Using the brightness constancy assumption, the equation for calculating the pixel flow velocity is described by (1).

(1) $$I\left(x,\;y,t\right)=I\left(x+\Delta x,\;y+\Delta y,\;t+\Delta t\right)=I\left(x,\;y,t\right)+\frac{\partial I}{\partial x}\Delta x+\frac{\partial I}{\partial y}\Delta y+\frac{\partial I}{\partial t}\Delta t+\varepsilon$$

I(x,y,t)—point (*x*, *y*) intensity function;
Δ*x*, Δ*y*—displacement of point between successively recorded frames;
Δ*t*—interval of time (between successively recorded frames);
*t*—time;
ε—higher-order terms of Taylor expansion.

Omitting higher-order terms represented by ε Equation (1) is simplified to the form (2). Next, after subtraction I(x, y, t) from both sides of Equation (2), the form (3) is achieved:

(2) $$I\left(x,\;y,t\right)\approx I\left(x,\;y,t\right)+\frac{\partial I}{\partial x}\Delta x+\frac{\partial I}{\partial y}\Delta y+\frac{\partial I}{\partial t}\Delta t$$

(3) $$\frac{\partial I}{\partial x}\Delta x+\frac{\partial I}{\partial y}\Delta y+\frac{\partial I}{\partial t}\Delta t=0$$

Equation (3) can also be rewritten (after division by Δ*t*) to the form (4).

(4) $$\frac{\partial I}{\partial x}\frac{\Delta x}{\Delta t}+\frac{\partial I}{\partial y}\frac{\Delta y}{\Delta t}+\frac{\partial I}{\partial t}=0$$

Equation (4) makes it possible to extract the expressions related to the pixel velocities *u* and *v* (in the *x* and *y* axes respectively) as well as s spatiotemporal derivatives of pixel brightness (5).

(5) $$I_{x}=\frac{\partial I}{\partial x},\;I_{y}=\frac{\partial I}{\partial y},\;I_{t}=\frac{\partial I}{\partial t},\;u=\frac{\Delta x}{\Delta t},\;v=\frac{\Delta y}{\Delta t}$$

*I_x_, I_y_*—brightness derivatives (spatial);
*I_t_*—brightness derivative (time);
*u*—horizontal velocity of pixel (flow);
v—vertical velocity of pixel (flow).

The relation (6), obtained on the basis of the presented considerations, makes it possible to determine the object’s normal speed in accordance with the direction of the gradient’s intensity.

(6) $$I_{x}u+I_{y}v=-I_{t},$$

The Horn–Schunck method was used in the detection algorithm to determine the optical flow. It assumes [37,38]:

- The intensity of a particular point is constant over time;
- Smoothness constraint (the neighboring points on an object have a similar optical flow).

These assumptions are met for almost all objects in the airspace that move outside the immediate vicinity of the vision system (the immediate vicinity means the distance at which intruders are not allowed to come closer). The problem could be detecting aircraft at night or in low light, when aircraft anticollision lights drastically change their brightness. Two objects moving side by side and overlapping each other can also be a problem (e.g., flight of planes in formation). These kinds of limitations are expressed by the smoothness of the flow field (7).

(7) $$\nabla^{2}u+\nabla^{2}v=\frac{\partial^{2}u}{\partial x^{2}}+\frac{\partial^{2}u}{\partial y^{2}}+\frac{\partial^{2}v}{\partial x^{2}}+\frac{\partial^{2}v}{\partial y^{2}}$$

To speed up numerical calculations, Laplacian values can be estimated using dependence (8). Parameters $\bar{u}$ and $\bar{v}$ are the mean results for the flow around a given point for the respective spatial components *x* and *y*.

(8) $$\nabla^{2}u=\Delta u=\bar{u}-u\;\nabla^{2}v=\Delta v=\bar{v}-v$$

According to the Horn–Schunck method, optical flow is defined as a global energy function (9), which should be minimized [40,41]:

(9) $$E=\iint \left[{\left(I_{x}u+I_{y}v+I_{t}\right)}^{2}+a^{2}\left({\left\|\nabla u\right\|}^{2}+{\left\|\nabla v\right\|}^{2}\right)\right]dxdy$$

A weighting factor for the smoothness constraint (higher values–smoother flow).

Solving Euler–Lagrange equations functional (9) can be minimized, and the output equations are linear (10). Two equations, presented in a matrix form, enable both the components of optical flow velocity to be determined at each point of the image.

(10) $$\left[\begin{array}{cc} I_{x}^{2}+a & I_{x}I_{y}\\ I_{x}I_{y} & I_{y}^{2}+a \end{array}\right]\left[\begin{array}{c} u\\ v \end{array}\right]=\left[\begin{array}{c} a^{2}\bar{u}-I_{x}I_{t}\\ a^{2}\bar{v}-I_{x}I_{t} \end{array}\right]$$

The Equation (10) can be ultimately transformed to the iterative Gauss–Seidl form (11), which is very useful in practice. Operations with the iterative dependencies significantly reduce the required computing power.

(11) $$u^{k+1}={\bar{u}}^{k}-\frac{I_{x}\left(I_{x}{\bar{u}}^{k}+I_{y}{\bar{v}}^{k}+I_{t}\right)}{I_{x}^{2}+I_{y}^{2}+a^{2}}\;v^{k+1}={\bar{v}}^{k}-\frac{I_{y}\left(I_{x}{\bar{u}}^{k}+I_{y}{\bar{v}}^{k}+I_{t}\right)}{I_{x}^{2}+I_{y}^{2}+a^{2}}$$

**Figure 1:**
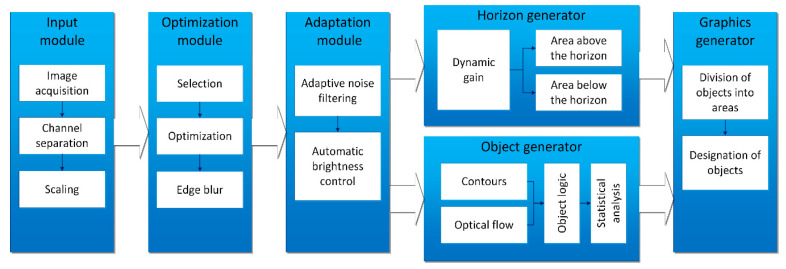
The structure of image processing algorithms.

Figure 1 shows a block diagram of the image processing algorithm. Its essential component is the detection algorithm using the optical flow method described above. In particular, this algorithm consists of the following blocks:

- The input module responsible for image acquisition from the camera and preprocessing;
- The optimization module used to adjust the image for further processing (e.g., depending on the time of day);
- Adaptive module where image filtering operations take place to eliminate disturbances caused by unstable lighting conditions. The example methodology is described in publication [32];
- The object generator module is the main part of this algorithm based on the optical flow method and the dynamic object contour extraction. The optical flow block generates the moveable background, then the contour block separates objects that have higher velocities than the background. Optical flow is the basis of the algorithm for detecting moving objects (see Figure 1). Its main task is to identify all moving points, which is done by thresholding the image of the square of the amplitude of velocity vectors calculated by the Horn–Schunck method. During the flight, all objects in the image usually move, including those that are static ground objects. So, it is necessary to eliminate them. This task is performed by the contour block, which masks the background from optical flow and separates potential intruder objects. The optical flow block and dynamic contour blocks ensure that only the objects with speed or size changes different than the moving background are selected. The output of these blocks is a binary image;
- The horizon generator block generates the binary mask image of the horizon, which facilitates object selection above and below the horizon line;
- The statistical block is used to count and label detected objects in the binary output image by connected components analysis. It calculates and accumulates some quantity and quality information about the binary representation of the detected objects on images;
- The graphics generator block generates graphical markers and inscriptions informing about the states of detected objects. It overlays this generated graphical information onto the actual image.

**Figure 2:**
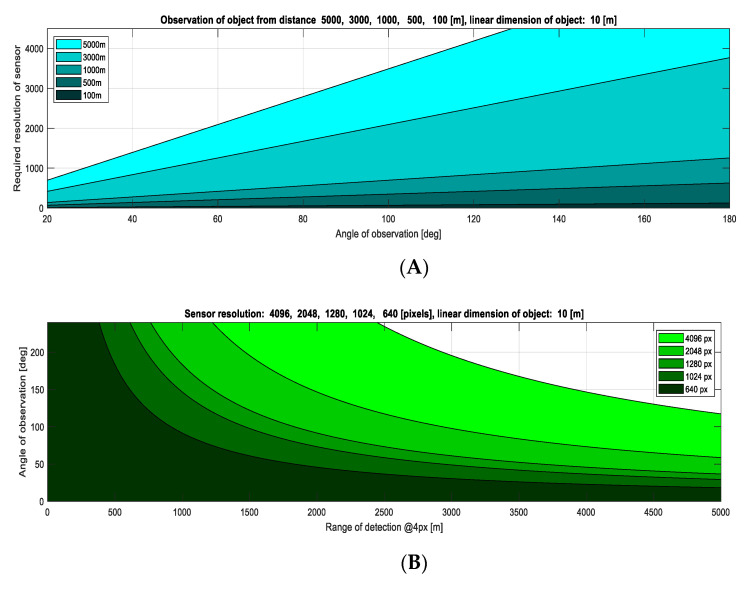
Dependence of sensor-required resolution on angle of observation and distance (**A**), and dependence on angle of observation on range of detection and resolution (**B**); simulations for linear dimension of object LDO = 10 m.

In the presented design of the anticollision vision system, it was assumed that the minimum resolution of the sensor would be 640 points, and the maximum 4096 points (horizontally). It was also assumed that it would be possible to detect an object from a minimum of four points arranged in a line. These assumptions were verified analytically and in real simulations [42] as well as in ground tests. The relationship between sensor resolution *n*, observation angle α as well as distance *d* and linear dimension of the detected object *LDO* is described by the Formula (12), which was derived in the work [42]. This formula can also be transformed due to α into the form (13). The value of *LDO* equals 10 m is representative for the wingspan of sports airplanes, small general aviation aircraft, MALE class UAVs, or the length of a typical sailplane. The objects of such *LDO* can be observed in the form of at least four points in one line from a maximum distance of 5 km, using a 4k sensor with angle observation limited to 117 deg (Figure 2). Using a 2k or HDTV1080p standard, 16:9 ratio for resolution 1920 × 1080, the maximal angle of observation is limited to 58 deg for equivalent conditions (*d* = 5 km, *LDO* = 10 m). By reducing the resolution, this angle is reduced correspondingly to 35 deg for HDTV720p standard, 16:9 ratio for resolution 1280 × 720, and 18 deg for VGA standard (still being considered, e.g., for infrared observation). Lower resolution image sensors are not taken into consideration. The calculations were also performed for objects of other linear dimensions and are presented in the work [42].

(12) $$n=\frac{4\times \alpha }{2\times atan\left(\frac{LDO}{2\times d}\right)}$$

*LDO*—linear dimension of object,
*d*—distance to object,
α—angle of observation.

(13) $$\alpha =\frac{n\times atan\left(\frac{LDO}{2\times d}\right)}{2}$$

The developed algorithms were the subject of comprehensive laboratory tests, including the use of advanced dedicated flight simulators [29,42,43] in the beginning, and then in-flight tests.

## 3. Preparation for Flight Tests

**Figure 3:**
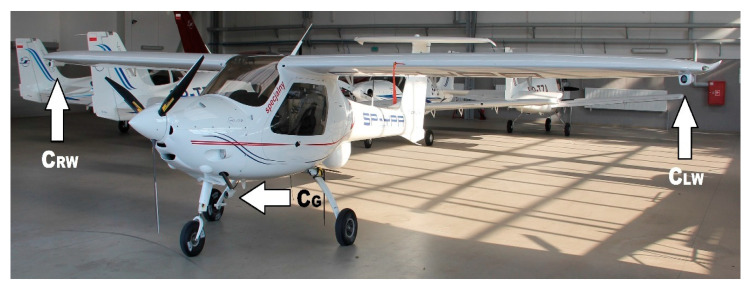
General view of the Czajka MP-02 airplane with an indication of the location of the observation sensors (C_RW_—right wing camera, C_LW_—left wing camera, C_G_—camera installed on the chassis).

For in-flight testing, an MP-02 Czajka airplane owned by the Department of Avionics and Control at the Faculty of Mechanical Engineering and Aeronautics of the Rzeszów University of Technology (Figure 3), was used. This is a general aviation class airplane operated as an OPV (optionally piloted vehicle). The aircraft was additionally equipped with a control system and an observation system originating from the LOT project [44].

According to the IDAAS project requirements, the manned airplane on which the reconnaissance sensors will be installed must be equipped with an on-board flight data recorder. The recorder is to ensure the recording of the following flight parameters:

- Geographical location;
- Ground speed;
- True track;
- Airspeed;
- Magnetic heading,
- Pressure altitude;
- GPS altitude;
- Climb rate;
- Airplane attitude.

The registration should cover the entire airplane mission (from the engine start to the engine stop). The avionics installed on board the MP-02 include the integrated Dynon Avionics D700 display system, which allows the registration of the required flight parameters and navigation data as well as the operating parameters of the power unit with a frequency of 4 Hz in the emergency recorder mode, and with a frequency of up to 16 Hz in the user programmed mode. The MP-02 equipment in the LOT version also includes the distributed measuring system PRP-W2 [45]. This system, in its full configuration, consists of the following elements:

- PCDL-01-data logger;
- PCDA-01-Air Data Computer (ADC);
- PCAH-01-Attitude and Heading Reference System (AHRS);
- PCAI-01-analog input module;
- PCDI-01-digital input module;
- PCGP-01-satellite navigation receiver module (GPS);
- PCIM-01-inertial quantity measurement module (IMU).

The modules are fully configurable, enabling the recording of flight parameters with a frequency of up to 1 kHz in the case of the PCIM-01 module. For the purposes of the research, the PCDA-01, PCAH-01 and PCGP-01 modules were configured for recording with a frequency of 50 Hz. The PRP-W2 system is not a limitation to possible applications on board small, unmanned aircraft due to its small dimensions, weight, and modular structure. For example, the largest dimension of the ADC module is 33 mm, the GPS is 35 mm, and the data logger is 27 mm.

**Figure 4:**
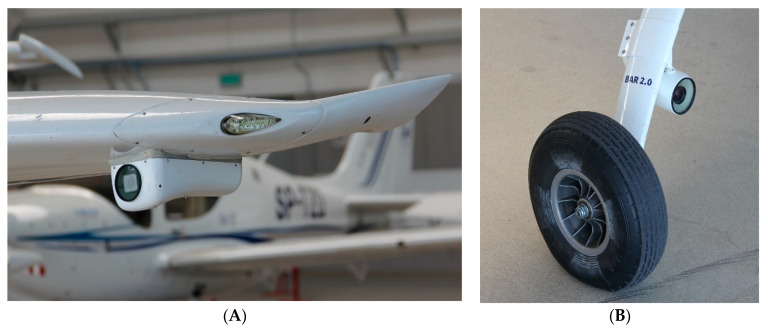
Observation sensor mounted in container under left wing (**A**); and camera installed on the right chassis (**B**).

Observation sensors were mounted on the airframe in dedicated containers. Two cameras were placed inside wing containers on the underside of the wing, while one camera was mounted on the main right gear (Figure 3 and Figure 4). During flight tests, images were recorded on flash disk drives, and video streams were processed directly on-board by the IDAAS computer.

## 4. Formal Requirements and Methodology for In-Flight Testing

### 4.1. Legal Conditions

During tests with the use of manned aircraft, the minimum safety distance requirements must be met. The flights were carried out under VFR (visual flight rules) conditions, so the pilots had to maintain a safe distance between aircraft. The tests were carried out in the airspace where VFR flights are allowed (class B, C, D, E, F, G spaces). Actually, tests were performed up to flight 95 (approx. 2900 m). This applies to both manned and unmanned aircraft. Therefore, space G, airport controlled zones (CTR), and airport controlled areas (TMA) should be taken into account. In a class G space, IFR (instrumental flight rules) and VFR flights are allowed, no separation is provided, but flight information service is provided. VFR flights can be performed with flight visibility of not less than 5 km. In particular, daily VFR flights at FIR (flight information region) Warsaw can be performed in an uncontrolled space from 30 min before sunrise to 30 min after sunset, in the altitude range at and below 900 m (3000 ft) AMSL (average medium sea level) or 300 m (1000 ft) above the ground—whichever is higher. Formal requirements for VFR are contained in the document [46]. Night research flights are not planned at this time.

### 4.2. Defining the Purpose of the Research

The objective of the test campaign was to conduct several functional tests of the observation system in terms of the image processing algorithm (IPA). These tests were both quantitative (differences in IPA operation on the aircraft relative to the results of ground tests and laboratory tests), and qualitative (the ability to detect objects depending on the distance, type of object, visibility, and lighting conditions). The main master purpose of the campaign was to conduct IPA efficacy tests:

- In selected air traffic situations;
- In various meteorological conditions and in various lighting conditions.

Comparative tests were carried out under:

- CAVOK conditions (Ceiling And Visibility OK) and small cloud cover (0/8 to 3/8);
- Light haziness and/or dustiness, and the absence of cloud cover or light cloud cover (0/8 to 3/8);
- Partial cloud (4/8 to 6/8);
- Complete or almost complete cloud (7/8 to 8/8).

Air traffic situations, object classes, and distances at which intruders can be detected were determined as a result of these tests. The situations when the detection of intruders by means of IPA or generally understood optical detection methods were difficult or impossible using the tested technologies were also found.

Basic functional tests were performed after installing (or placing) the required equipment on board the aircraft, which consisted of starting the hardware and software and checking the correct functioning of the system under test. The minimum scope of the check was:

- Operation of IPA in a qualitative sense by generating movement in the field of view of the camera/cameras;
- Correct recording of video material and information generated by IPA;
- Correct operation of the data recording system.

In case of any divergences between laboratory tests and flight test results, their causes needed to be identified. The required corrections or clarifications should then be made before the next stage of the tests. After obtaining positive static test results, the performance of 2–3 circular flights or one short (several minutes) flight in the airport area was performed. During this flight, moving ground objects were within the range of the observation system cameras. If possible, the flight was carried out under a cloudless sky or with a sufficiently high uniform cloudiness, without clearly outlined cloud shapes. During and after this flight, the following were qualitatively verified:

- Correct recording of video material and information generated by IPA;
- Correct operation of the data recording system.

Quantitative analysis included:

- Phenomena related to the possible false detection of intruders as moving objects against the sky/clouds;
- Phenomena related to the detection of real as well as false moving objects against the background (or on the ground);
- Correctness of the horizon line detection process and distinguishing between objects placed above and below the horizon.

### 4.3. Basic Flight Scenarios

**Figure 5:**
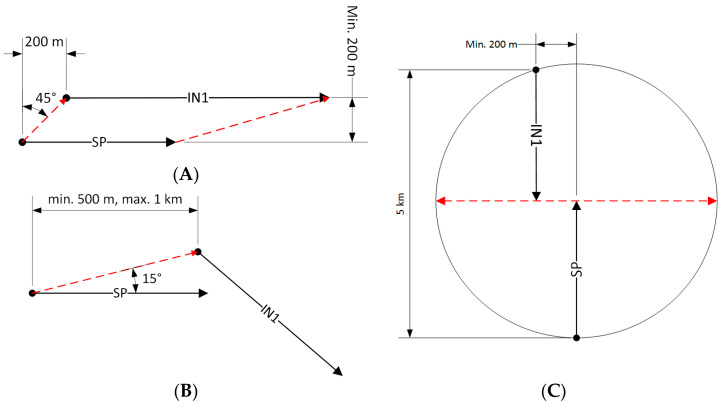
Intruder detection scenario on a parallel course (**A**); intruder detection scenario on a collision course, convergent value courses (**B**) and opposite courses (**C**); IN1—intruder number 1, SP—IDAAS equipped airplane.

In three basic scenarios, the flights of aircraft equipped with the IDAAS system (object marked as SP) were parallel (Figure 5A), convergent (Figure 5B) and opposite (Figure 5C) to the courses of an intruder (object marked as IN1).

**Figure 6:**
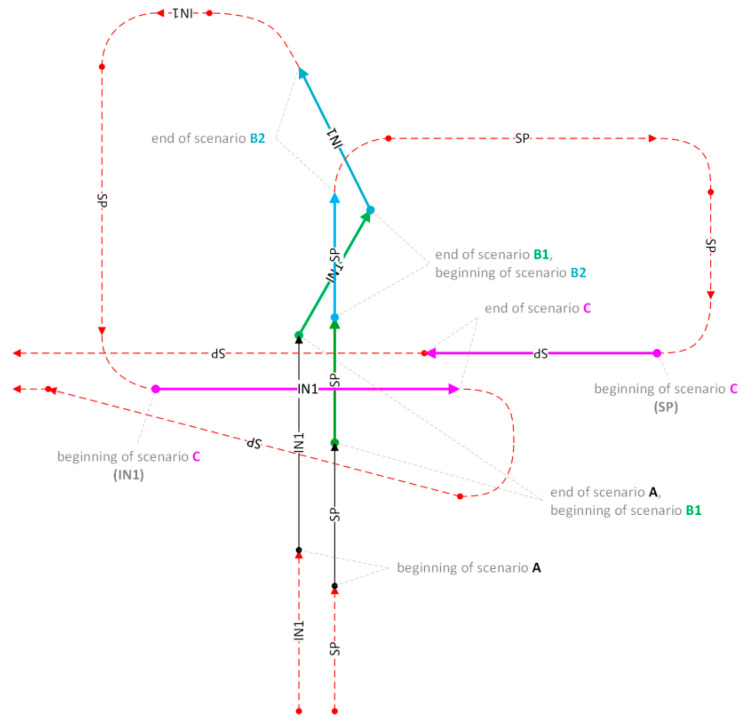
Graphical diagram of the flight plan for implementing CS-23/CS-25 class intruder detection scenarios; IN1—intruder number 1, SP—IDAAS equipped airplane.

In scenario A (Figure 5A), it was assumed that the intruder would fly with TAS (true air speed) approximately 20 kt faster than SP. The scenario started when the intruder was visible at a 45-degree angle to the left of the SP (Figure 6, beginning of scenario A). The test was finished when IN1 was at an angle of 15 degrees to the left side of the SP (Figure 6, end of scenario A).

In the second scenario (Figure 5B) the intruder IN1 crossed an SP’s trajectory from left to right at the front of it. The scenario started when the intruder was at an angle of 15 degrees to the left front side of the IDAAS aircraft (Figure 6). Both aircraft maintained courses until an intruder position of 45 degrees to the right side was reached. Next, the intruder started passing from the right side to the left side.

The third scenario started when both aircraft flew on opposite courses with lateral offset of a minimum of 200 m (Figure 5C)—for safety reasons. The scenario finished when the aircraft passed each other. The modification of these scenarios were maneuvers in which the aircraft flew at constant but different altitudes.

In the second modification, there was more than one intruder. For instance two CS-23/25 class aircraft or a glider, hand-glider, paraglider instead of one of them. Other cases are not described in detail in this work, rather they are an attachment to the IDAAS project documentation.

All three scenarios were laid in A, B1, B2, C order creating the test flight plan. All of them were defined so that the end of the preceding scenario became the beginning of the next, or simple maneuvers of both aircraft were necessary to start the next scenario. In this way, the flight trajectory was optimized to reduce the number of not useful legs (Figure 6).

## 5. Selected Elements of the In-Flight Tests

**Figure 7:**
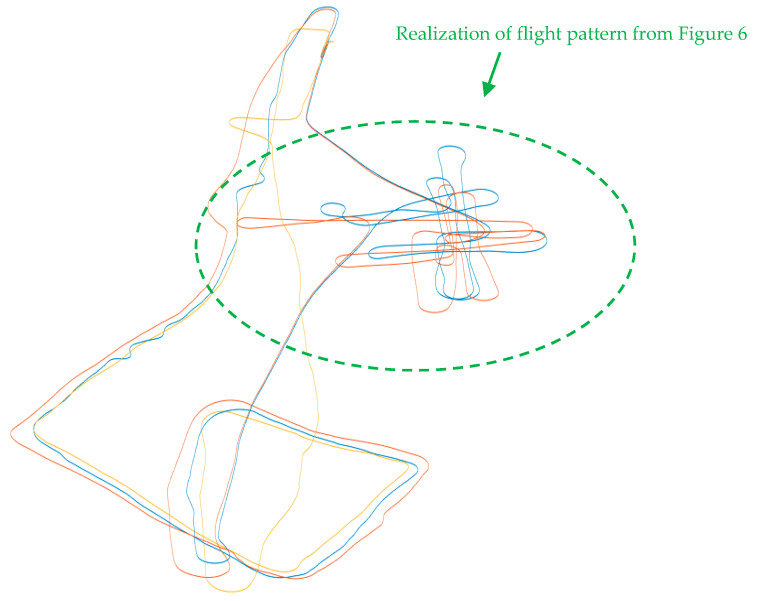
Flight route realized on 13 October, 2018 according to the diagram presented in Figure 6 (SP—blue, IN1—red, IN2—yellow).

The flight plan presented in Section 4.3 was implemented in different atmospheric and lighting conditions and for various combinations of airplane altitudes. This section presents examples of the results obtained during these flights. In Figure 7, the real flight route realized by SP and IN1 is shown according to the diagram presented in Figure 6. During the SP flight, an additional IN2 object entered into the test airspace.

**Figure 8:**
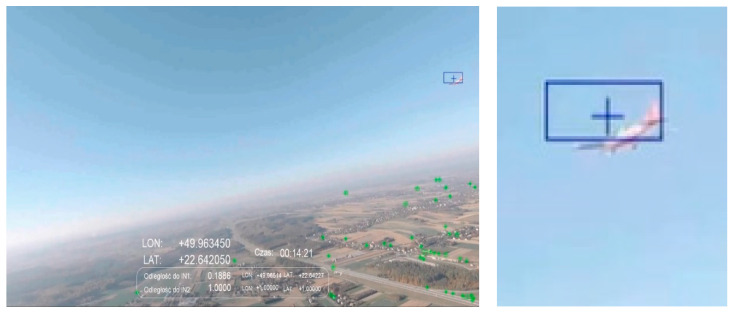
Scenario related to Figure 5A; afternoon, sun ~120 degrees to the left, altitude ~300 m; distance to intruder: 188 m.

Figure 8 shows the case in which an IN1 intruder was detected flying 188 m from SP. This is an example of the detection of an ultralight aircraft with a span of 11.8 m. The flight was on a convergent course with the intruder according to the scenario presented in Figure 5A. A photo was taken approximately at noon at a height of 300 m above ground level. Detection of the object against a background of a cloudless sky was performed flawlessly. Unfortunately, false object markers appeared against the ground (objects above the horizon are marked with a blue marker, and those set against the ground are marked with a green marker). This problem was observed especially during low altitude flights with very good air clarity.

The disturbances observed in these conditions, due to the large number of false indications against the ground (reaching even several dozen in the worst cases), make the detection system useful for the airspace observed above the horizon only.

**Figure 9:**
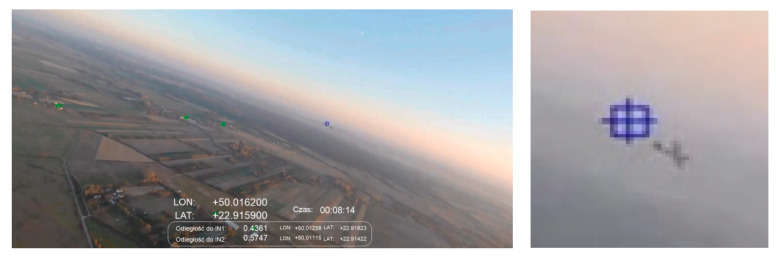
Scenario related to Figure 5C; evening, sun ~90 degrees to the right, altitude ~200 m; distance to intruder: 436 m.

**Figure 10:**
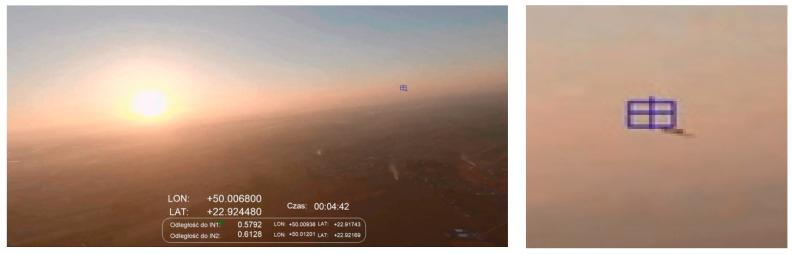
Scenario related to Figure 5C; sunset, altitude ~500 m, distance to intruder: 579 m.

**Figure 11:**
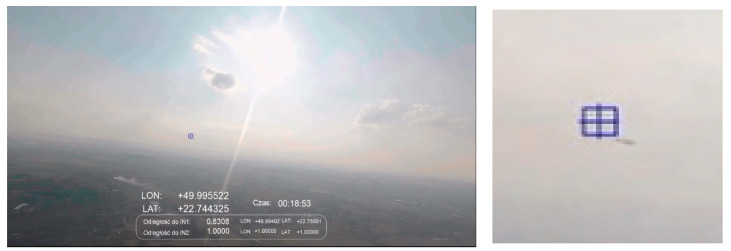
Scenario related to Figure 5B; late afternoon, altitude ~500 m, distance to intruder: 830 m.

**Figure 12:**
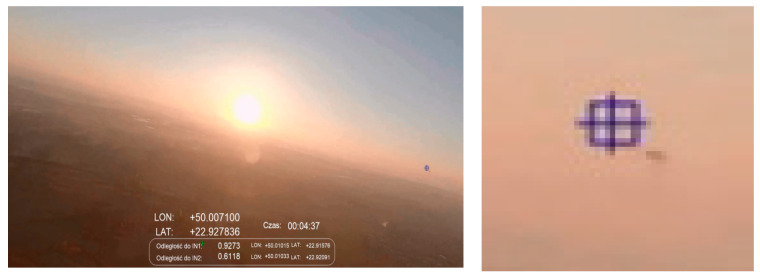
Scenario related to Figure 5B; sunset, altitude ~500 m, distance to intruder: 927 m.

**Figure 13:**
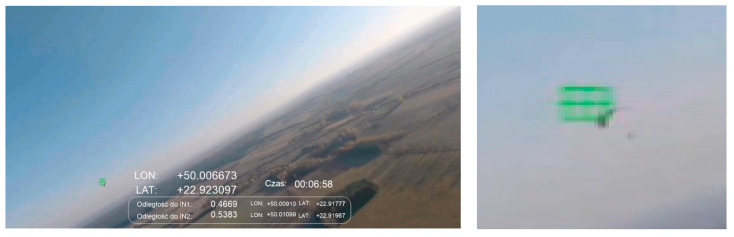
Detection of paraglider; afternoon, sun ~90 degrees to the right, altitude ~150 m, distance to intruder: 466 m.

Figure 9 is a shot taken half an hour before sunset. The sun disc is approximately 120 degrees to the right of the object, the detected intruder is visible almost exactly on the horizon line. The object (this is the same airplane as visible in Figure 8) is much further away than in the Figure 8 scenario, but was detected correctly. In this case, only sporadic problems related to the detection of false objects against the ground were observed (mainly variable reflections of light coming from buildings on the ground). Incorrect indications appeared as short-lasting flashes at intervals of several seconds. This type of disturbance can be easily removed in the future by introducing additional low-pass filtering of the resulting data. The blue intruder marker is located slightly to the left and above the intruder. It was deliberately and artificially moved by the authors of the software. An offset was introduced so as not to obscure the object. This action was applied to Figure 8, Figure 9, Figure 10, Figure 11, Figure 12 and Figure 13.

Figure 10 shows an example of detecting the same type of intruder as presented in Figure 9, but from a distance of 579 m. In this case, IN1 is approximately 20 degrees to the right of the setting solar disc. Figure 9 presents the detected object located almost exactly on the horizon line, while in Figure 10 the intruder is visible just above the horizon (it flew higher than the aircraft equipped with the IDAAS sensor). The shot was taken during sunset, against the background of a cloudless sky. In these conditions, the tested algorithm did not detect false objects against the sky and within the horizon. Occasional and short-term false alarms appeared from variable light reflections on the ground (mainly reflections from water surfaces—rivers, lakes, ponds) but they can be removed in future research by introducing additional low-pass filtering of the output data.

The flights shown in Figure 9 and Figure 10 follow the scenario from Figure 5C—aircraft on opposite courses. Due to the influence of many external factors, e.g., cooperation efficiency between the crews and test operators or temporary traffic situations, it was not always possible to execute the flight plan perfectly. Especially in Figure 9, we can see that the planes were moving on opposite courses but were in a turn, correcting their relative position and increasing separation.

The shots shown in Figure 10, Figure 11 and Figure 12 were taken at an altitude of over 500 m. In these cases, false alarms related to the detection of ground objects were already rare (single and impermanent, their lifetimes were tenths of a second), and the detection of IN1 was efficient even if it was located at a distance of about 1 km (Figure 12).

Figure 13 shows an example of the automatic detection of a paraglider passing 466 m from the SP. The detected object was clearly visible above the horizon. The sun was about 90 degrees to the right of the frame and remained quite high above the horizon (the flight was made in the late afternoon). In these conditions, the detection of the intruder was almost flawless. Fake object markers on the ground appeared sporadically and briefly. The actual intruder was detected continuously and efficiently.

**Figure 14:**
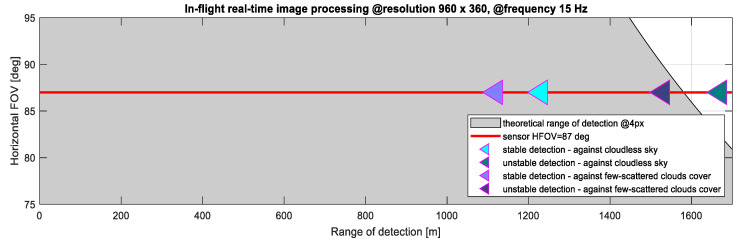
Qualitative analysis of the detection of an intruder with LDO = 10 m (results for image processing on board a flying plane, real time computations).

Figure 14 presents selected quantitative results of the analyses concerning the effectiveness of intruder detection (LDO = 10 class). These results relate to real-time image processing in flight conditions. They show that the developed algorithm working on a dedicated hardware platform enabled the stable detection of intruders against a cloudless sky or scattered clouds up to a distance of about 1200 m. Detection of intruders is possible up to distances exceeding 1600 m, but the obtained results are not always stable. The results obtained for the intruders against the ground were similar to the observations against clouds, however, due to numerous disturbances (false detections), they were not included in Figure 14 (it should be emphasized that false detections in such cases decrease significantly with increasing altitude).

**Figure 15:**
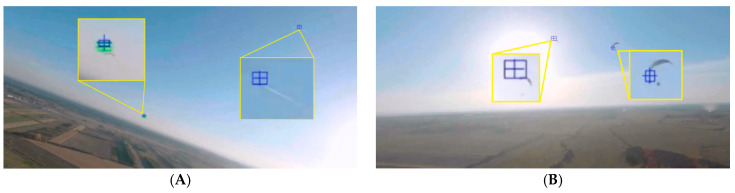
Situations of multiple object detection: paraglider and airliner (**A**), two paragliders (**B**).

The intruder detection algorithm was also tested in situations of the simultaneous appearance of multiple objects (intentional tests or accidental situations). In Figure 15A, we can see both the paraglider at a distance of about 1 km, but also an airliner flying at cruising altitude, which accidentally appeared in the field of view of the camera. The intended test of the possibility of simultaneous detection of two paragliders is presented in Figure 15B. The paragliders were detected at distances of approx. 300 m and 800 m. In both of these cases, the flight took place at an altitude of about 150 m above farmland and under a cloudless sky. Under such conditions, the algorithm worked properly.

## 6. Discussion

The qualitative and quantitative analysis of the obtained results shows that the false detection of objects in airspace can occur. The implemented and tested algorithm was very sensitive to disturbances caused by low clouds which moved against a distant background (the effect of their absolute as well as relative movement caused by observer flight). During the flights at a low altitude in very good visibility, the algorithm also generated false alarms for objects under the detected horizon line. Paradoxically, in the best meteorological conditions with very good visibility, the number of false indications against the background increased. False readings below the horizon were numerous (reaching even several dozen in the worst cases), although relatively unstable (blinking). The operation of the system in such conditions can be improved by introducing an additional output data filtration system. The intruders detected in the same examples over the horizon line were very persistent, and there were no false alarms in this area of the analyzed image. During flights at an altitude of more than 500 m above the ground, false alarms occurred sporadically as short-lasting flashes at intervals of several seconds (including objects above and below the horizon). In the tests performed, the horizon line detection process and distinguishing between objects placed above and below the horizon were correct, in general. Problems with the correct detection of the horizon by optical algorithms were observed, among others, when the sun disk was located near the edge of the image. This problem, of course, also applied to cases where the real horizon was not visible.

The paper presents the results of an algorithm that worked in real time on the on-board computer. In the literature, we can find many articles related to the detection of intruders using vision methods, but only a few present preparation, planning, implementation and analysis of flight test results [20,47,48]. In this article, special attention was paid to the proper preparation and planning of comprehensive tests of the vision anticollision system. The proposed detailed scenarios and their integration into a complex flight plan made it possible to check the operation of the vision system in various lighting conditions during one flight.

The tests performed in laboratory conditions, which preceded the flight tests, were realized on hardware with higher computing performance. The results obtained in laboratory conditions [42], as well as the postflight analysis realized on efficient computing stations, indicated significantly greater possibilities of the proposed image processing algorithm (in relation to the on-board real-time processing results discussed in this paper). The postflight analyses showed that the algorithms were much less susceptible to generating false alarms (this is related to the possibility of image processing with higher frequency and resolution). Short-term (tenths of a second) false alarms, if anything, happened in “postprocessing” processes at intervals of several seconds at most.

Future work will be directed towards both improving the data processing algorithm itself and achieving greater computing performance by the on-board computer. Another conclusion that comes from the obtained flight tests is the need to improve the filtering system for output data from the image analysis and detection algorithms. A properly selected and tuned filtration system will make it possible to extinguish the markers of unstable objects, appearing sporadically against the background, especially during flights at low altitude in very good visibility. In the next steps, it is considered that equipping the algorithm with an object classifier would be advantageous because it is not possible to detect the type of intruder using the present version of the algorithm. It would also enable the autonomous determination of the approximate distance to the intruder (assuming a known LDO, we can try to estimate it), even without the need to use stereovision or the cooperation of other measurement systems. The steps to be taken in the future also include research with the use of various optical sensors, including cameras operating in various infrared ranges.

## Data Availability

The data presented in this study are available on request from the corresponding author.
